# Mucous Polypi in the Nose

**Published:** 1914-11

**Authors:** John C. Warbrick

**Affiliations:** Chicago


### Mucous Polypi in the Nose.

By John C. WARBRICK, M. D., Chicago.

Polypi of the mucous variety are supposed to be derived from the mucous membrane of some part of the nasal passages or from some condition of it that tends to predispose to them.

They are about the most common growth of innocence to be found in the nostrils occurring almost at any period of life from a few years up to old age, being no respecter of persons but found in both men and women alike and among persons of all nationalities. They would seem to arise from some place in the upper part of the nasal cavity mostly and then to extend downwards towards the floor of the nose and either forwards or backwards as conditions in the cavity favor and more so when they are attached by a long pedicle as they often are. Where the passage is narrow and the polypus of any size it may be squeezed in between the surfaces and in time flattened to fit itself in and assume the shape of the passage while it becomes of firmer consistence from the constant pressure.

Polypi are almost more certain to form when catarrhal conditions are present and there is any obstruction of the nostrils, but this may not always be the case, but many times it is the rule. Mucous polypi then are supposed to arise from the upper part of the nasal cavity, from the roof of the nostril or from the middle turbinated body to some extent and other places as well, but in my experience they do not seem to take their origin any more from the middle turbinated body than from other places while some authorities give it as the principal place from which they arise. This may be due to the fact that its pedicle takes origin from a point high up in the nostril and the body of it is always in a pendent position and when enlarged it is likely to become pressed against adjacent structures not an uncommon thing, thus obstructing the mucous.

From the fact that the pedicle of the middle turbinated body has its origin high up in the nostril, it might be supposed that mucous polypi could arise from it much more readily than elsewhere, and more so when catarrhal conditions exist.

It is somewhat of a common thing to find the middle turbinated body diseased in some way so it may become a polypoid mass itself, or a bag from degeneration, in fact the whole of it may form a pus bag or a small part of it only. The body may be inflamed and shed pus or a good deal of mucous, and if it contains pus it may split open and allow it to escape, while this condition may be bilateral or unilateral, the mucous membrane over the middle turbinated body would seem to have a tendency to degenerate especially in some individuals when it is not well supplied with blood for a number of reasons, more so in later years when the circulation is more likely to get sluggish and when exercise is a necessity, but not the rule, consequently catarrhal conditions are often brought on which in time affect the mucous membrane. Mucous polypi seldom if ever seem to arise from the inferior turbinated body, possibly on account of its length and low position, but they may do so. It is somewhat common, however, to be able to see mucous flowing from about the middle or posterior part of the lower turbinate on to the floor of the nasal cavity, and this seldom or never occurs with the middle turbinated body, although when diseased it may shed both mucous and pus, but the functions of the two bodies are different.

One may be said to shed its mucous from time to time, while that of the other in most cases being unable to do so, may sooner or later form poylpi. One hangs by a pedicle perpendicular, while the other projecting is horizontal.

One has the function of contracting and expanding, while the other has not.

One is low down in the nostril, while the other is high up.

Polypi of the soft variety are supposed to be dilations of the mucous membrane or parts of it occurring in certain places.

Mucous polypi while they may derive their origin from some parts of the nasal cavity, more than from others, may arise from almost any part of it where there is mucous membrane.

They may arise from the septum almost anywhere, but it is not a common thing for them to form very much on that surface.

They may arise from almost any part of the outer wall of the nasal cavity from the front to the back, either in single or multiple numbers or in small clusters. Mucous polypi it would seem may form in the nostrils to such an extent they may almost block up both cavities completely.

In one case I removed about one hundred and seventy-five polypi from a woman's nose in size varying from a pin head to a twenty-five cent piece. In another case about eighty-five were removed from a man's nose, some of them being large in size.

Mucous polypi may be of various shapes, such as oval, flat, oblong, round, according to their position and condition in the nasal cavity. Their size may be anyway from a pins head up to that of a twenty-five cent piece or larger, and of a different shape. The number found in the nostrils may be single or multiple, and from one up to two hundred or more.

Mucous polypi it would seem are affected to some extent by atmospheric conditions. When the weather is damp and wet they seem to swell and enlarge more and make their presence more noticed, but when the weather is warm this does not occur so much. It may be said that mucous polypi seldom occur in strong, healthy, vigorous persons, with all their body functions in good working order, but they might do so.

